# Scented Solutions: Examining the Efficacy of Scent Interventions in Mitigating Driving Fatigue

**DOI:** 10.3390/s24082384

**Published:** 2024-04-09

**Authors:** Xinyue Jiang, Kanesan Muthusamy, Jian Chen, Xueliang Fang

**Affiliations:** 1 Department of Mechanical Engineering, Faculty of Engineering, Technology and Built Environment, UCSI University, Kuala Lumpur 56000, Malaysia; tcfangxueliang@163.com; 2 Department of Electronic Engineering, Yangzhou Technical Vocational College, Yangzhou 225000, China; 3 Department of Mechanical Engineering, Yangzhou University, Yangzhou 225127, China; jian.chen@yzu.edu.cn; 4 College of Traffic Engineering, Yangzhou Polytechnic Institute, Yangzhou 225127, China

**Keywords:** scent, fatigued driving, fatigue identification, EEG, fatigue countermeasure

## Abstract

Fatigued driving threatens the safety of people’s lives and property. Scent countermeasures offer minimal disruption and high efficacy, making them a promising approach. The aim of this study was to explore the application of scent countermeasures in alleviating fatigued driving. This study explored changes in EEG frequency bands (alpha, beta, theta, and gamma) and the activity of EEG metrics (*R*_(*α*/*β*)_, *R_θ_*_/(*α*+*β*)_ and *R*_(*α*+*θ*)/(*α*+*β*)_) in the temporal lobe during driving tasks, selected fatigued driving identifiers, and aided validation by investigating subjective fatigue with the Karolinska Sleepiness Scale (KSS). The EEG indicators all increased, with a significant increase in *R*_(*α*/*β*)_. *R*_(*α*/*β*)_ was combined with the KSS to explore the effects of three scents, peppermint, grapefruit, and lavender, on driving fatigue. The subjective questionnaire results indicated that all three scents significantly improved driving fatigue, with significantly lower levels of driving fatigue compared to the control group. The analysis of EEG signals revealed a significant decrease in *R*_(*α*/*β*)_ after the implementation of scent countermeasures. Moreover, *R*_(*α*/*β*)_ was found to be lower in all three odor intervention groups compared to the control group. All three scents were found to significantly alleviate driving fatigue. The grapefruit scent had a better timely effect in relieving driving fatigue and the lavender scent had a longer effectiveness. This study provides further exploration for the application of odor interventions to alleviate driving fatigue. This study provides a practical reference for drivers to use odors to avoid fatigue in order to improve road safety.

## 1. Introduction

According to the World Health Organization, the global annual number of road traffic fatalities reached 1.35 million in 2016 and continues to rise steadily [1]. Driver fatigue has been identified as a contributing factor in nearly 20% of road accidents and 25% of fatal and serious injuries [2]. The U.S. National Highway Traffic Safety Administration reports that between 56,000 and 100,000 accidents caused by drowsiness occur each year, resulting in over 1500 deaths and 71,000 injuries [3].

The implementation of effective methods and techniques to alleviate fatigue is crucial for safeguarding driver safety and improving road traffic safety. Numerous studies have proposed various strategies to prevent and mitigate fatigue, including music [3,4], temperature regulation [5,6], lighting, and caffeine [7], among others. The analysis of experimental data revealed that olfactory stimuli were more effective in enhancing the subjects’ attention levels compared to auditory stimuli [8]. Olfactory intervention has emerged as a noteworthy method for combating fatigue due to its minimal disruption to regular activities and work, rendering it a promising approach.

In recent years, several studies have investigated the effects of odors on fatigued driving. Pujiartati et al. conducted a simulated driving experiment under three conditions and found increased awareness with both short and prolonged exposure to peppermint odor [9]. Mahachandra et al. demonstrated that peppermint can help maintain driver alertness by examining the slope of the electroencephalographic (*α* + *θ*)/*β* ratio [10]. Funato et al. investigated scent presentation techniques for drivers and developed a system that simulated an actual vehicle cabin to test the stimulating effects of scent on drivers and its effective duration [10]. Hirata’s study identified effective methods for releasing odors to maintain alertness [11]. Yoshida et al. investigated the effects of four fragrances, including peppermint, rosemary, eucalyptus, and lemon, on combating driving drowsiness [12]. Tang et al. explored the arousal effects of peppermint, lemon, and lavender gas odors at various concentrations on driving fatigue [1].

However, there are some issues in previous studies on odor intervention for fatigued driving. Drivers were exposed to the odor for the entire duration of the study, which may have led to olfactory fatigue [11], potentially limiting the effectiveness of the odor intervention. The study was conducted using a simple driving simulator in an indoor environment, which differs significantly from real-world scenarios in terms of spatial size and driving conditions. The accuracy of fatigued driving studies with odor intervention may be influenced by the research environment.

In response to the aforementioned issues, this study constructed a simulated driving platform based on real vehicle environments, making the research environment more authentic and the results more reliable. The odor intervention experiment protocol was optimized to maximize the effectiveness of odor intervention. Through simulated driving experiments, drivers’ EEG activity was examined and indicators to characterize fatigued driving were determined. Subsequently, the fatigued driving indicators were combined with subjective fatigue questionnaires, and three odors (peppermint, grapefruit, and lavender) were intervened after driver fatigue to explore their effects on driving fatigue.

## 2. Study on Fatigued Driving Recognition

The EEG is the golden indicator for accurately identifying fatigue driving [13,14] and serves as the basis for this study. In investigating the fatigue-alleviating effects of scent on driving, the meticulous selection of indicators characterizing fatigued driving becomes paramount. Utilizing simulated driving experiments, EEG indicators that reliably signify fatigue in drivers are systematically chosen, establishing a fundamental framework for subsequent research on scent intervention in fatigued driving.

### 2.1. Materials and Methods

#### 2.1.1. Participants

We enlisted participants, randomly choosing 14 male subjects with ages ranging from 20 to 26 years. The average age of all participants was 24.71 ± 0.99. It is noteworthy that the majority of car accidents resulting from fatigued driving tend to happen among the younger demographic [15]. All subjects possessed a valid driving license and were in sound health. In the 48 h leading up to the experiment, they abstained from consuming stimulant-rich diets containing alcohol, caffeine, or other stimulants, as well as stimulant medications. Prior to the experiment, participants ensured they had received ample sleep and were in a positive mood.

#### 2.1.2. Experimental Equipment

**Figure 1:**
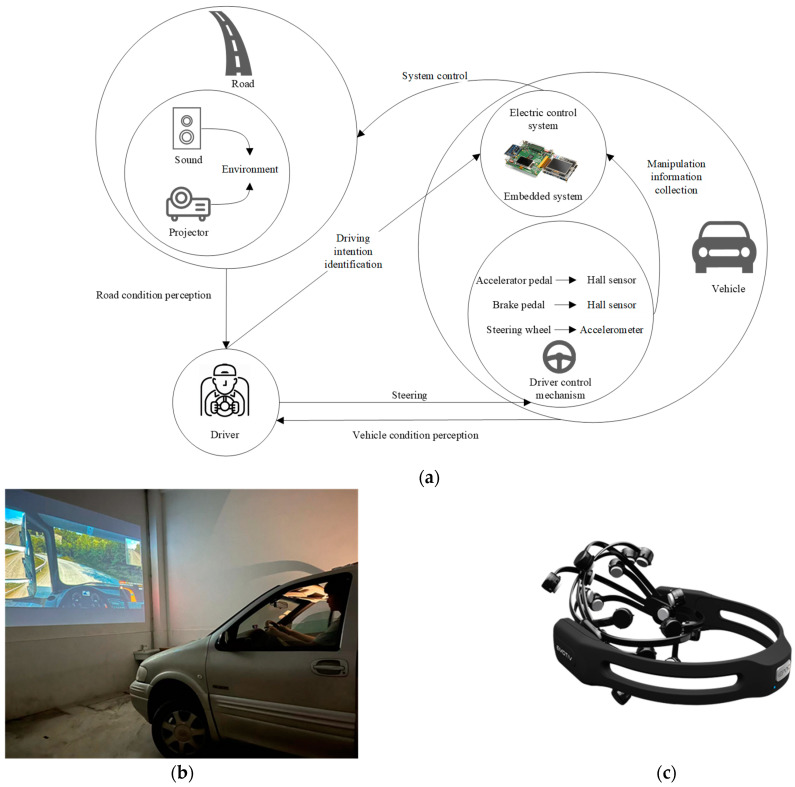
(**a**) Driving simulation platform system architecture; (**b**) driving simulation platform; (**c**) EMOTIV Epoc+.

This study utilized the simulated driving platform depicted in Figure 1a,b. The Buick Commercial Vehicle GL8 serves as the simulated cockpit environment, and the essential functionalities of the initial simplistic automobile driving simulator were integrated into the actual vehicle. The primary board and display system of the driving simulator were preserved, and an embedded control system is incorporated. Hall sensors were employed to gather data from the throttle and brake pedals. The six-axis accelerometer captures the steering wheel angle signal, and the embedded system converts the signal to align with the output of the original steering wheel. The traffic environment was emulated using a projector and sound.

For the collection of driver EEG signals, we employed the EMOTIV Epoc+ 14-channel wireless portable EEG instrument manufactured by Emotiv Inc., headquartered in San Francisco, CA, USA, as illustrated in Figure 1c.

Subjective driver fatigue was assessed using the Karolinska Sleepiness Scale (KSS) [16]. This nine-point scale ranges from one to nine, where one represents extreme wakefulness and nine signifies extreme drowsiness. Previous research has established a correlation between KSS scores and collision risk in both simulated driving studies and real-road driving studies [17].

#### 2.1.3. Experimental Conditions

The experimental sessions were scheduled between 2:00 p.m. and 4:00 p.m. to align with the typical occurrence of fatigued driving during the mid-afternoon “siesta” period associated with increased sleepiness [15]. The laboratory maintained a temperature range of 15~25 °C. The road traffic environment was deliberately set to be monotonous, featuring a sunny day with vehicles traveling on a motorway bordered by trees on both sides. Subjects were instructed to drive at a speed of 80–100 km/h without overtaking, as monotony in the driving environment is known to heighten the susceptibility to fatigue [18].

#### 2.1.4. Procedure

The entire experiment spanned approximately 1 h per participant, following the outlined procedure. Participants initially acquainted themselves with the experimental setup and underwent a 5 min adaptation period for simulated driving. Subsequently, they donned an EEG signal acquisition device. Participants completed a subjective fatigue questionnaire. The EEG signal acquisition commenced as participants engaged in a 30 min simulated driving task. It’s noteworthy that continuous driving for 20–30 min in a monotonous environment induces driver fatigue [19]. Upon completion of the driving task, the signal acquisition equipment was removed and participants filled out the subjective fatigue questionnaire once again.

### 2.2. Data Analyses and Results

Raw EEG signals from the temporal lobe region of each participant were imported into Matlab Online. To eliminate background signals and long-term drift, a 0.16 Hz first-order high-pass filter, tailored to the characteristics of the electronics, was applied.

Following the reduction of EEG signal noise, frequency domain analysis became imperative for comparison. Power spectral estimation, serving as the primary method, transforms the EEG—whose amplitude varies with time—into a spectrogram illustrating EEG power fluctuations across frequencies. This visualization aids in understanding the transformation and distribution of EEG rhythms. In this study, the direct method from classical spectral estimation theory was selected for analyzing the power spectral characteristics of EEG signals.

Simultaneously, EEG signals were processed within 1 min intervals. The average power spectral density of each frequency band, including *θ* (4–8 Hz), *α* (8–12 Hz), *β* (12–25 Hz), and *γ* (25–45 Hz) waves, was determined through integration, as exemplified by β waves in Equation (1).

In recent research, formulas such as Equation (*α*/*β*) [20], Equation (*θ*/(*α* + *β*)) [20], and Equation ((*α* + *θ*)/(*α* + *β*)) [21] have been employed as assessment metrics for fatigued driving. Calculate the per-minute EEG metrics *R*_(*α*/*β*)_, *R_θ_*_/(*α*+*β*)_, and *R*_(*α*+*θ*)/(*α*+*β*)_, as depicted in Equation (2).

(1) $$G_{\beta }=\frac{\int_{a}^{b}P(\omega )d(\omega )}{b-a}$$

(2) $$R_{\left(\alpha /\beta \right)}=G_{\alpha }/G_{\beta }$$

where, $G_{\beta }$ signifies the average power spectral density of a specific frequency band of *β* waves, *P*(*ω*) represents the EEG signal power spectral density, *b* denotes the upper limit of the *β* wave frequency band, and *a* signifies the lower limit of the *β* wave frequency band, *R*_(*α*/*β*)_ denotes the ratio of the average power spectral density between the *α* and *β* wave bands, with $G_{\alpha }$ representing the average power spectral density of a specific frequency band of the *α* wave.

The analysis involved segmenting each of the three EEG metrics, along with alpha, beta, theta, and gamma activities, into 2 min intervals throughout the driving task. Paired samples *t*-tests were conducted to compare selected EEG metrics at 5 min versus 30 min after the commencement of the driving task, aiming to identify significant differences in EEG metrics before and after the driving task. Mean values are presented, and a significance level of *p* < 0.05 was considered.

The KSS values obtained from the subjective fatigue questionnaire before and after the driving task underwent a paired samples *t*-test to examine significant differences in subjective fatigue pre- and post-driving task. Mean values are presented, and a significance level of *p* < 0.05 was considered.

**Figure 2:**
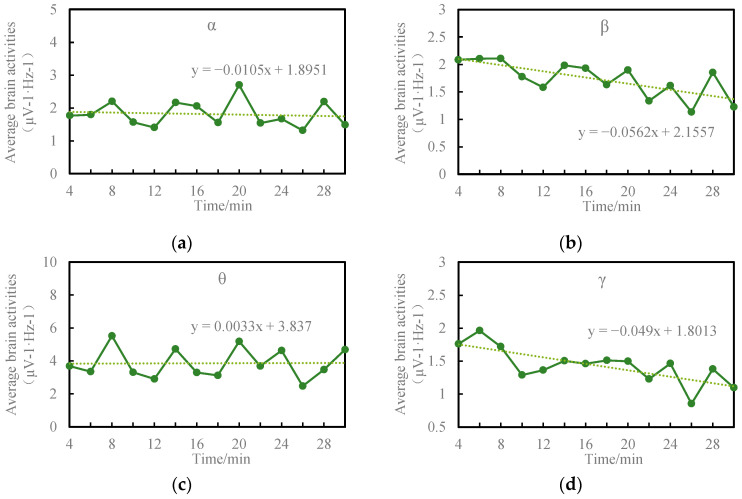
Average brainwave activity during the driving task. (**a**) Alpha activity; (**b**) beta activity; (**c**) theta activity; (**d**) gamma activity.

Figure 2 depicts the temporal evolution of alpha, beta, theta, and gamma activities throughout the driving task. Slow-wave EEG activity demonstrated a gradual increase, reflected in a slight rise in theta-wave activity. Conversely, fast-wave EEG activity exhibited a general decline over time, indicated by a slight reduction in alpha-wave activity, decreased gamma-wave activity, and a more pronounced decrease in beta-wave activity.

**Figure 3:**
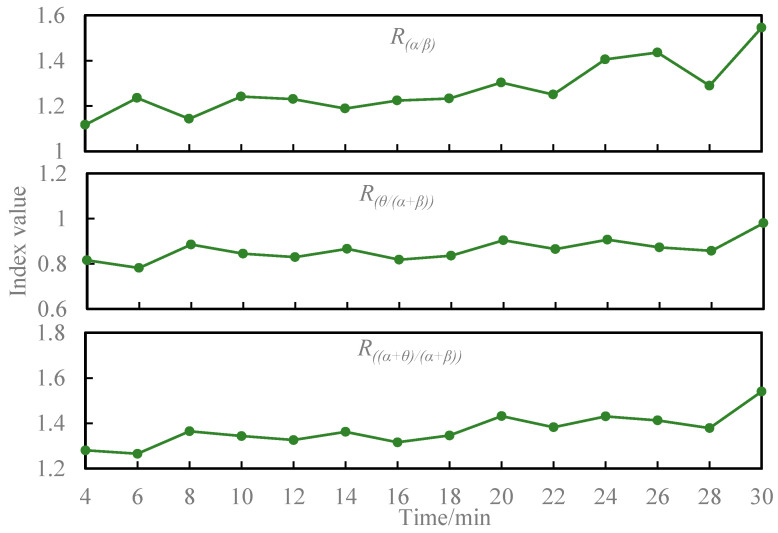
Average activity plotted over time during the driving task for EEG indicators.

As depicted in Figure 3, the EEG indices *R*_(*α*/*β*)_, *R_θ_*_/(*α*+*β*)_, and *R*_(*α*+*θ*)/(*α*+*β*)_ exhibited an increasing trend over time during the driving task. Upon evaluating the normality of the populations, it was observed that the mean differences for *R*_(*α*/*β*)_ (*p* = 0.895), *R_θ_*_/(*α*+*β*)_ (*p* = 0.400), *R*_(*α*+*θ*)/(*α*+*β*)_ (*p* = 0.549), and KSS (*p* = 0.163) were normally distributed. Table 1 presents the outcomes of the paired samples *t*-test comparing the three EEG metrics and KSS before and after the driving task. Significantly, *R*_(*α*/*β*)_ (*t* = −2.254, *p* = 0.042) and KSS (*t* = −5.292, *p* = 0.000) exhibited substantial increases after the driving task. *R_θ_*_/(*α*+*β*)_ and *R*_(*α*+*θ*)/(*α*+*β*)_ also demonstrated increased values post-driving.

*R*_(*α*/*β*)_ emerges as a robust identifier of driving fatigue, exhibiting more pronounced differences with extended driving time compared to other EEG indicators. Consequently, the EEG indicator *R*_(*α*/*β*)_ was integrated with the subjective fatigue questionnaire to investigate the impact of odor on driving fatigue.

## 3. Study on Scent Interventions for Fatigued Driving

In the experiment on scent intervention in fatigued driving, we gathered EEG signals and subjective fatigue scores KSS from drivers. We calculated the driving fatigue recognition index *R*_(*α*/*β*)_ under various odor interventions, including peppermint, grapefruit, and lavender. The mental state of drivers was analyzed during the intervention with these three types of odors to assess the effectiveness of each in alleviating fatigue during driving.

### 3.1. Materials and Methods

#### 3.1.1. Participants

The experiment involved 11 male volunteers aged between 20 and 26 years old. The average age was 24.55 ± 0.93 years. All participants were in good health, possessed a valid driver’s license, and refrained from consuming stimulants such as alcohol or caffeine 48 h before the experiment. Additionally, they had adequate sleep and were in a positive mood. Participants had a normal sense of smell, and none of them had received professional olfactory training. These 11 participants all took part in the fatigued driving identification experiment. The remaining three participants who had previously participated in the fatigued driving identification experiment were unable to participate in the current experiment due to reasons such as time constraints and physical conditions.

#### 3.1.2. Experimental Conditions

Peppermint is primarily composed of menthol, flavonoids, and non-flavonoid phenolic carboxylic acids [22]. Surveys indicate a preference for peppermint aroma [23]. Several studies have found that peppermint scent can help maintain driver alertness and have the most significant arousal effect on driver fatigue [1,9]. Citrus odors, such as grapefruit, can alleviate fatigue and improve task performance [24]. The scent of orange can reduce fatigue during exercise [25], while the scent of lemons can prevent fatigue while driving [1,12]. Research has shown that the scent of lavender can alleviate fatigue [26,27]. Lavender aroma has also been implicated in studies related to alleviating driving fatigue [1]. Therefore, peppermint, grapefruit, and lavender were selected for this study to explore their effects on alleviating driving fatigue.

Every volunteer underwent four distinct conditions, detailed as follows:

- Control Group: No scent intervention throughout the experiment;
- Peppermint Group: Peppermint scent intervention at the 30 min point of the experiment;
- Grapefruit Group: Grapefruit scent intervention at the 30 min point of the experiment;
- Lavender Group: Lavender scent intervention at the 30 min point of the experiment.

Hirata suggests that the optimal concentration for scent release is 10%, which generates a strong odor intensity level of 4 without causing any unpleasant experiences [11]. Additionally, he discovered that setting the release time to 60 s without fluctuation maximized the effect of maintaining alertness. Therefore, in this experiment, a 10% scent spray composed of AA Skincare essential oils, alcohol, and water in a ratio of 1:6:3 was used. The scent was released for 1 min when the drivers felt tired after driving for 30 min.

Experiments transpired on a simulated driving platform, with EEG signals recorded by EMOTIV Epoc+ and subjective driver fatigue assessed using KSS. The experimental timeframe was set from 2:00 p.m. to 4:00 p.m., and the laboratory maintained a temperature of 15~25 °C. The traffic scenario involved driving on a motorway flanked by trees on both sides on a sunny day. Throughout the driving task, participants were instructed to maintain a speed of 80–100 km/h without overtaking.

#### 3.1.3. Procedure

Participants received training on operating the experimental platform before the experiment commenced. At the start of the experiment, participants wore the signal acquisition equipment and familiarized themselves with the experimental procedure. Following 5 min of adaptation to the driving simulation, the EMOTIV Epoc+ began operation, and the experiment timer was initiated. At the 28 min mark of simulated driving, participants completed the KSS subjective fatigue questionnaire to self-assess their mental state at that juncture. When subjects reached 30 min of driving, the spray device released a scent spray 30 times for 1 min (or in the absence of scent intervention). Following the scent intervention, subjects continued the driving simulation until the conclusion of 45 min of driving. The EMOTIV Epoc+ was turned off, participants filled in the subjective fatigue questionnaire once more, the collection device was removed, and the experiment concluded.

### 3.2. Data Analyses and Results

#### 3.2.1. Subjective Fatigue Assessment Questionnaire KSS

Upon evaluating the normality of populations, it was determined that the KSS difference before and after odor intervention in the control group (*p* = 0.635), mint group (*p* = 0.255), grapefruit group (*p* = 0.231), and lavender group (*p* = 0.097) were within normal distribution. Post-intervention, KSS differences between the control and mint group (*p* = 0.494), control and grapefruit group (*p* = 0.293), and control and lavender group (*p* = 0.285) also exhibited normal distribution.

The KSS scores before and after the odor intervention were individually compared within the Control group, Peppermint group, Grapefruit group, and Lavender group using paired samples *t*-tests. Subsequently, the KSS scores after the odor intervention were compared between the control group and each intervention group separately. The results are presented as means, and significance was considered at *p* < 0.05.

**Figure 4:**
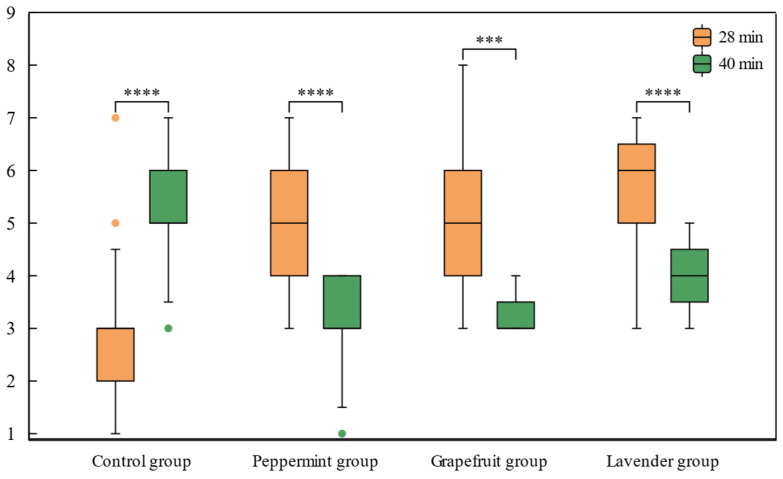
Boxplot for average KSS in each condition. Statistically significant differences are highlighted with *** *p* < 0.01; **** *p* < 0.001.

As depicted in Figure 4 and detailed in Table 2, KSS values exhibited a significant increase with driving time in the control group (*t* = −4.847, *p* = 0.001). In contrast, the peppermint group (*t* = 5.186, *p* = 0.000), grapefruit group (*t* = 3.684, *p* = 0.004), and lavender group (*t* = 5.190, *p* = 0.000) demonstrated significantly reduced KSS scores.

Table 2 indicates a noteworthy reduction in KSS within the Peppermint group (*t* = 4.353, *p* = 0.001), Grapefruit group (*t* = 5.244, *p* = 0.000), and Lavender group (*t* = 3.545, *p* = 0.005) in comparison to the KSS in the control group at the conclusion of the experiment.

Additionally, a one-way ANOVA was performed to assess the disparity in KSS before and after the odor intervention in the Peppermint group, Grapefruit group, and Lavender group. The results revealed no significant difference among the three groups (*p* > 0.05).

#### 3.2.2. EEG Indicator *R*_(_*_α_*_/_*_β_*_)_

Raw EEG signals from the temporal lobe region of each subject were preprocessed using Matlab. Subsequently, the fatigued driving recognition index *R*_(*α*/*β*)_ per minute was calculated.

**Figure 5:**
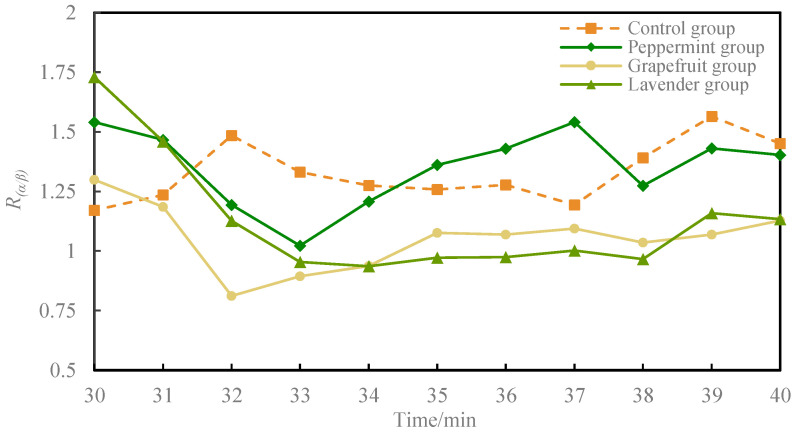
Mean *R*_(*α*/*β*)_ change curves for the four groups during 30 to 40 min.

The activity of the fatigued driving recognition index *R*_(*α*/*β*)_ was analyzed by dividing each 1 min interval after odor intervention, as shown in Figure 5. As the driving time progressed, the fatigued driving recognition index *R*_(*α*/*β*)_ significantly increased in the control group. In the peppermint group, *R*_(*α*/*β*)_ significantly decreased after the odor intervention, reaching its lowest point after 33 min and then increasing. In the grapefruit group, *R*_(*α*/*β*)_ decreased significantly after the odor intervention until 32 min, then increased slowly, remaining lower than that of the control group after 31 min. In the lavender group, *R*_(*α*/*β*)_ decreased significantly until 33 min, continued to decrease slowly until 34 min, and then increased slowly, remaining lower than the control group after 32 min. At the end of the experiment, *R*_(*α*/*β*)_ was lower in all three odor intervention groups than in the control group.

The *R*_(*α*/*β*)_ at 28 min for each group (peppermint group, grapefruit group, and lavender group) was compared with that at 32 min, 34 min, 36 min, 38 min, and 40 min, respectively, using a paired samples *t*-test. The results are reported as means and significance is considered at *p* < 0.05.

**Figure 6:**
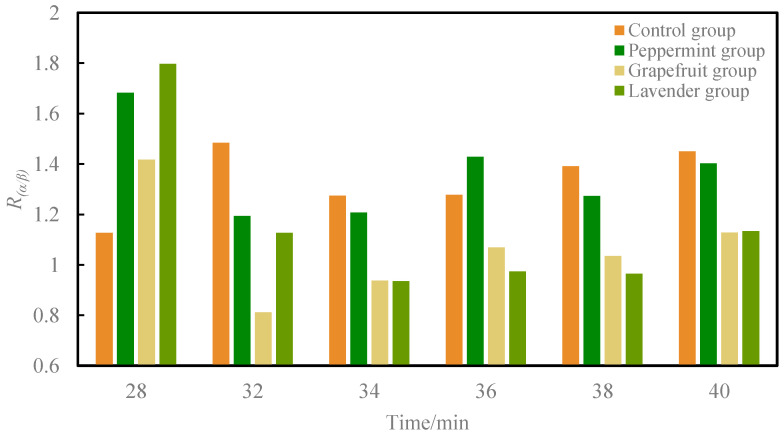
Column diagram comparing mean *R*_(*α*/*β*)_ in each condition.

Table 3 and Figure 6 illustrate that the peppermint group exhibited a decrease after odor intervention compared to pre-intervention, with 32 min (*t* = 2.273, *p* = 0.046) and 34 min (*t* = 2.561, *p* = 0.028) showing significant differences compared to pre-intervention. Similarly, the grapefruit group demonstrated a decrease after odor intervention compared to pre-intervention, with 32 min (*t* = 3.931, *p* = 0.003), 34 min (*t* = 2.924, *p* = 0.015), and 38 min (*t* = 2.505, *p* = 0.031) exhibiting significant differences compared to pre-intervention. In the Lavender group, after odor intervention, 32 min (*t* = 2.550, *p* = 0.029), 34 min (*t* = 3.790, *p* = 0.004), 36 min (*t* = 3.833, *p* = 0.003), 38 min (*t* = 4.018, *p* = 0.002), and 40 min (*t* = 3.106, *p* = 0.011) all showed significant decreases.

*R*_(*α*/*β*)_ at 32 min, 34 min, 36 min, 38 min, and 40 min in the peppermint, grapefruit, and lavender groups were subjected to repeated measures ANOVA. The within-group effect was analyzed to determine whether there was a significant change in the EEG indicator *R*_(*α*/*β*)_ over time under the influence of peppermint, grapefruit, and lavender odor interventions. The between-group effect was analyzed to ascertain if there was a significant change in the EEG index *R*_(*α*/*β*)_ under different odor types. The level of significance was set at *p* < 0.05. The results indicated a statistically significant interaction between the treatment effect and time effect (*F* = 2.050, *p* = 0.017), no statistically significant treatment main effect (*F* = 1.176, *p* = 0.331), and a statistically significant time main effect (*F* = 4.905, *p* = 0.002).

## 4. Discussion

Fatigued driving is one of the major causes of traffic accidents, significantly impacting road traffic safety.

In this investigation, we initially explored EEG activities in the temporal lobe regions across four frequency bands (alpha, beta, theta, and gamma) through a fatigued driving experiment. Based on these bands, we derived four EEG indicators: *R*_(*α*/*β*)_, *R_θ_*_/(*α*+*β*)_ and *R*_(*α*+*θ*)/(*α*+*β*)_. All EEG metrics are presented as ratios of EEG activity. Guo et al. proposed Equation (*α*/*β*) [20], and Jap et al. proposed Equation (*θ*/(*α* + *β*)) [28] and Equation ((*α* + *θ*)/(*α* + *β*)) [21], which can be employed as potential tools for fatigue detection.

This study observed a slight rise in theta wave activity and a decline in alpha, gamma, and beta wave activity with prolonged driving time.

Higher levels of fast-wave activities (alpha and beta) are typically associated with alertness [29]. Previous research indicates that alpha wave activity decreases as fatigue sets in during extended driving [21,28]. Increased beta activity is linked to heightened alertness and attention [30], while studies suggest a decline in beta wave activity when drivers feel drowsy [31,32,33]. Belyavin and Wright [14] assert that a sharp drop in beta wave activity is a key indicator of reduced alertness, consistent with the findings of this study. Additionally, Han et al. reported a significant decrease in beta and gamma rhythm during states of fatigue [34].

Theta wave activity, known to correlate with human fatigue, tends to increase when an individual is fatigued [17,30,35,36]. Research on driver fatigue has consistently demonstrated an elevation in theta wave activity during fatigued driving episodes [31,37].

In this investigation, we observed a consistent increase in the EEG metrics *R*_(*α*/*β*)_, *R_θ_*_/(*α*+*β*)_, and *R*_(*α*+*θ*)/(*α*+*β*)_ as driving time progressed. A notable difference in the EEG metric *R*_(*α*/*β*)_ was evident before and after the driving task.

This aligns with the findings of Jap et al., who reported an increase in Equation (*α*/*β*) with extended driving time [21]. Eoh et al. demonstrated a decreasing trend in the (*β*/*α*) ratio as driving fatigue increased [31]. The trend observed in the present study, with a reversal and an increasing trend in the electroencephalographic index *R*_(*α*/*β*)_, echoes these findings.

Furthermore, Jap et al. suggested a significant difference in temporal lobe area activity for Equation (*θ*/(*α* + *β*)) during monotonous driving in train drivers. The mean values for both frontal and temporal lobe site activity were reported to be significantly different for Equations (*θ*/(*α* + *β*)) and ((*α* + *θ*)/(*α* + *β*)) [28].

Following the initial investigation, an odor intervention fatigued driving experiment was conducted. This experiment integrated the subjective fatigue questionnaire KSS with a driving fatigue indicator, *R*_(*α*/*β*)_, to assess the impact of three odors-peppermint, grapefruit, and lavender on driving fatigue.

After the peppermint odor intervention, subjective fatigue, as assessed by KSS, significantly decreased and remained significantly lower compared to the control group at the end of the experiment. Additionally, the EEG indicator *R*_(*α*/*β*)_ showed a significant decrease within 4 min after the intervention compared to the pre-intervention period.

Peppermint, known for its essential oils primarily consisting of menthol, flavonoids, and non-flavonoid phenolic carboxylic acids, has been associated with reducing fatigue and enhancing alertness during monotonous work settings [22]. Surveys indicate that people prefer the scent of peppermint among essential oils such as peppermint, rosemary, and basil. Pujiartati et al. conducted simulated driving experiments utilizing EEG, HRM, and KSS, revealing increased alertness in participants exposed to peppermint odor compared to those without the scent, regardless of the duration of exposure [23]. Mahachandra et al. demonstrated that the slope of the (*α* + *θ*)/*β* ratio was lower in the group exposed to peppermint freshener compared to the placebo group, suggesting peppermint’s potential to maintain driver alertness when used as an in-vehicle fragrance [9]. Tang et al. investigated the effects of peppermint, lemon, and lavender odors, as well as their concentrations, on physiological signals such as ECGs, PPGs, RESPs, and the Stanford Drowsiness Scale. They found that an 80% concentration of peppermint gas had the most pronounced arousal effect on driving fatigue [1].

After the intervention with grapefruit scent, subjective fatigue, as measured by KSS, significantly decreased, and by the end of the experiment, it remained significantly lower compared to the control group. The EEG indicator *R*_(*α*/*β*)_ also exhibited a decrease, with significant differences observed at 32 min, 34 min, and 38 min compared to pre-intervention.

Citrus odors have been associated with fatigue alleviation and improved task performance [24]. Studies have shown that citrus aurantium essential oil can alleviate anxiety and fatigue in myocardial infarction patients [25]; the scent of orange can reduce fatigue during exercise [38]; and the aroma of sweet orange can alleviate fatigue after engaging in activities such as playing with blocks [39]. Similarly, the scent of lemons has been found to sustain alertness and prevent fatigue while driving [1,12].

After the intervention with lavender scent, subjective fatigue as measured by KSS decreased significantly, and by the end of the experiment, it remained significantly lower compared to the control group. Both EEG indicators *R*_(*α*/*β*)_ also exhibited significant decreases within 10 min after the intervention.

Lavender has been traditionally used as an analgesic in massage therapy and inhalation therapy [40]. Studies have highlighted lavender as a treatment for sleep problems [41]. Additionally, research has demonstrated that lavender scent can alleviate fatigue, as evidenced by its efficacy in reducing fatigue in surgical technicians [26] and cardiac patients [27]. Moreover, studies have found that lavender scent can effectively alleviate driving fatigue [1].

This study compared the effects of several highly rated odors on fatigued driving, which to some extent fills a gap in the research field. Moreover, odor intervention strategies mitigate the fatigue properties of olfaction. By constructing a simulated driving platform based on actual vehicles for simulated driving experiments, the study replicates realistic driving environments, thereby improving the accuracy of the research results.

We recognize the complexity of factors that influence fatigued driving. Studies have highlighted the impact of various factors such as driver age, gender [15], psychological factors [42], driving experience [43], sleep status [44], work hours [2], and environment [18] on fatigued driving. Studies have indicated that olfactory abilities differ by gender [45,46]. The effect of the same odor can also vary between different genders [47]. Therefore, the next step will be to account for the influences of fatigue on driving and to investigate whether they have a moderating effect on odor interventions for fatigued driving. We will further investigate the effects of odor intervention strategies (e.g., concentration, duration, and frequency) and factors such as odor preference on fatigued driving to gain a more comprehensive understanding of odor interventions for fatigued driving. This will increase the validity and applicability of our findings in the field of fatigued driving interventions.

## 5. Conclusions

Fatigued driving can lead to traffic accidents, affecting road traffic safety and causing injuries and property damage. It is necessary to implement effective measures to alleviate fatigued driving, and olfactory measures represent a promising approach. This study delves into the application of odor intervention to mitigate fatigue during driving.

Initially, the activities of four EEG frequency bands (alpha, beta, theta, and gamma) in the temporal lobe area, along with three EEG indicators (*R*_(*α*/*β*)_, *R_θ_*_/(*α*+*β*)_ and *R*_(*α*+*θ*)/(*α*+*β*)_), were investigated through simulated driving experiments. Fatigued driving recognition indicators were selected through paired-sample *t*-test comparisons, and subjective fatigue was assessed using the KSS for auxiliary validation. Following the driving task, there was an increase in *R_θ_*_/(*α*+*β*)_ and *R*_(*α*+*θ*)/(*α*+*β*)_), accompanied by significant rises in *R*_(*α*/*β*)_ and KSS.

Subsequently, *R*_(*α*/*β*)_ was combined with KSS to explore the effects of three odors—peppermint, grapefruit, and lavender—on driving fatigue through an odor intervention experiment. The results of the subjective questionnaire showed that all three odors, peppermint, grapefruit, and lavender, caused a significant decrease in all levels of driving fatigue, as well as a significantly lower level of driving fatigue compared to the control group. Moreover, the fatigued driving EEG recognition index demonstrated that all three odors significantly mitigated driving fatigue. The type of odors did not show a significant effect on fatigued driving, but there was a statistically significant interaction between the treatment effect and the time effect. Grapefruit odor exhibited more immediate relief of driving fatigue, whereas lavender odor showed longer-lasting effectiveness.

Our study provides valuable insights into the potential application of odor intervention as a method to mitigate fatigue during driving, aiming to explore optimal odor intervention strategies for enhancing road safety and reducing the risks associated with fatigued driving.

## Figures and Tables

**Table 1:** Paired *t*-test result for the difference between the pre- and post-task of *R* and KSS.

Variables	Pre-Task	Post-Task	Diff	*t*	*p*
*R*_(*α*/*β*)_	1.129	1.425	−0.296	−2.254	0.042
*R_θ_*_/(*α*+*β*)_	0.826	1.061	−0.235	−1.896	0.080
*R*_(*α*+*θ*)/(*α*+*β*)_	1.300	1.588	−0.288	−2.131	0.053
KSS	2.929	4.929	−2.000	−5.292	0.000

Diff, difference (pre- minus post-task); *t* = *t*-ratio (shows the deviation of the difference between the means); *p* = *p*-value (specifies significance level).

**Table 2:** Paired *t*-test result of KSS in each condition.

Group	28 min		40 min		28 min/40 min			Control Group 40 min/40 min		
	M	SD	M	SD	Diff	*t*	*p*	Diff	*t*	*p*
Peppermint group	5.000	1.342	3.091	0.944	1.909	5.186	0.000	2.182	4.353	0.001
Grapefruit group	5.000	1.483	3.273	0.467	1.727	3.684	0.004	2.000	5.244	0.000
Lavender group	5.727	1.191	4.000	0.775	1.727	5.190	0.000	1.273	3.545	0.005
Control group	3.000	1.673	5.273	1.104	−2.273	−4.847	0.001	-	-	-

**Table 3:** Paired *t*-test result for difference between pre- and post-intervention of *R*_(*α*/*β*)_ in each condition.

Group	32 min	34 min	36 min	38 min	40 min
Peppermint group	**2.273**	**2.561**	0.802	1.317	0.990
	**(*p* = 0.046)**	**(*p* = 0.028)**	(*p* = 0.441)	(*p* = 0.217)	(*p* = 0.346)
Grapefruit group	**3.931**	**2.924**	2.175	**2.505**	1.676
	**(*p* = 0.003)**	**(*p* = 0.015)**	(*p* = 0.055)	**(*p* = 0.031)**	(*p* = 0.125)
Lavender group	**2.550**	**3.790**	**3.833**	**4.018**	**3.106**
	**(*p* = 0.029)**	**(*p* = 0.004)**	**(*p* = 0.003)**	**(*p* = 0.002)**	**(*p* = 0.011)**

## Data Availability

All data included in this study are available upon request by contact with the corresponding author. The data are not publicly available because of ethical restrictions.
